# Double-Faced Immunological Effects of CDK4/6 Inhibitors on Cancer Treatment: Challenges and Perspectives

**DOI:** 10.3390/bioengineering11111084

**Published:** 2024-10-29

**Authors:** Yongqin Liu, Yiying Deng, Chang Yang, Hua Naranmandura

**Affiliations:** 1 Department of Public Health, Zhejiang University School of Medicine, Hangzhou 310058, China; 2 Department of Hematology of First Affiliated Hospital, Zhejiang University School of Medicine, Hangzhou 310058, China; 3 Cancer Center, Zhejiang University, Hangzhou 310058, China

**Keywords:** CDK4/6 inhibitor, immunomodulatory effect, drug resistance, immune microenvironment, immunotherapy

## Abstract

Cyclin-dependent kinases (CDKs) are generally involved in the progression of cell cycle and cell division in normal cells, while abnormal activations of CDKs are deemed to be a driving force for accelerating cell proliferation and tumorigenesis. Therefore, CDKs have become ideal therapeutic targets for cancer treatment. The U.S FDA has approved three CDK4/6 inhibitors (CDK4/6is) for the treatment of patients with hormone receptor-positive (HR^+^) or human epidermal growth factor receptor 2-negative (HER2^−^) advanced or metastatic breast cancer, and these drugs showed impressive results in clinics. Besides cell-cycle arrest, there is growing evidence that CDK4/6is exert paradoxical roles on cancer treatment by altering the immune system. Indeed, clinical data showed that CDK4/6is could change the immune system to exert antitumor effects, while these changes also caused tumor resistance to CDK4/6i. However, the molecular mechanism for the regulation of the immune system by CDK4/6is is unclear. In this review, we comprehensively discuss the paradoxical immunological effects of CDK4/6is in cancer treatment, elucidating their anticancer mechanisms through immunomodulatory activity and induction of acquired drug resistance by dysregulating the immune microenvironment. More importantly, we suggest a few strategies including combining CDK4/6is with immunotherapy to overcome drug resistance.

## 1. Introduction 

Cell cycle is the series of events regulating duplication of DNA and cell division to produce two daughter cells [1]. However, the dysregulation of cell cycle could result in abnormal cell proliferation, leading to hyperactivation of downstream signaling pathways that result in sustained proliferation, which is closely related to the occurrence and development of cancers [2,3]. Generally, cyclin-dependent kinases (CDKs), especially CDK4 and CDK6, play a vital role in regulating the G1/S transition of cell cycle through cell-cycle checkpoints and transcriptional events in response to extracellular and intracellular signals [4]. Therefore, CDK4 and CDK6 are thought to be attractive targets for certain cancer therapies.

Over the past three decades, the U.S. Food and Drug Administration (FDA) has approved three specific CDK4/6 inhibitors (CDK4/6is), namely, palbociclib, ribociclib, and abemaciclib, for the treatment of postmenopausal women with estrogen receptor-positive (ER^+^) or human epidermal growth factor receptor 2-negative (HER2^−^) advanced or metastatic breast cancer in combination with endocrine therapy, which has now become the standard first-line therapy [5,6]. Mechanistically, CDK4/6is can selectively block cell-cycle progression, leading to the inhibition of cell proliferation, tumor cell senescence, and the disruption of energy metabolism. In addition, these inhibitors are currently being extensively tested in other types of tumors to expand their indications, including lung cancer, pancreatobiliary cancers, mesothelioma, chronic lymphocytic leukemia, and so on [2,7,8,9]. In particular, several emerging preclinical models have explored the synergistic effects of CDK4/6is with other agents (e.g., cisplatin, adavosertib, and taxol), aiming to achieve promising clinical efficacy with an acceptable toxicity profile [6].

Although CDK4/6is have achieved great success in clinics, drug resistance (particularly acquired resistance) is becoming a big challenge [7,8]. Moreover, increasing evidence has indicated that CDK4/6is not only have an anticancer effect but also promote tumor immune escape, which might contribute to drug resistance [9]. Notably, it has recently been suggested that CDK4/6is are also able to alter the tumor microenvironment (TME) [10], which is suggested to play a double-faced role in patients treated with CDK4/6is. These significant findings could serve as a roadmap for future research on immune modulation to overcome CDK4/6i resistance [11].

In this review, we discuss in depth the essential role of CDK4/6is and their anticancer effects brought about by increased tumor cell immunogenicity, activation of effector T cells, induction of memory CD8^+^ T cells, and immunogenic cell death. Moreover, we also summarize the alteration in the immune microenvironment contributing to acquired drug resistance, which is also a major challenge in clinics. Additionally, here we suggest a few promising clinical approaches like combining these inhibitors with immunotherapy to overcome drug resistance.

## 2. Cancer Therapeutic Strategies Targeting CDK4/6 Cyclins

### 2.1. The Function of CDK4 and CDK6 Kinases in Normal and Malignant Cells

Generally, in normal cells, cell cycle is a highly ordered series of events consisting of four sequential phases (i.e., G1, S, G2, and M), and is tightly regulated by the expression and activation of various mitogenic factors, which trigger cascades of intracellular signaling networks [4]. In particular, CDK4/6 is a type of highly homologous serine/threonine kinase that is commonly involved in cell-cycle regulation [12,13].

During cell cycle, D-type cyclins are essential partners of CDK4/6 utilized for their kinase activity and they act as a regulatory subunit of cyclin-dependent kinases [14]. Upon adequate mitogenic stimulation, CDK4 and CDK6 kinases form complexes with D1-type cyclin to drive cell-cycle progression from the G1 to S phase through the following major processes [15]. Briefly, the CDK4/6–cyclin D1 complex initially mediates the activation of retinoblastoma family members, including RB, p107, and p130. Subsequently, RB dissociates from E2F transcription factors, which increases E2F transcriptional activity, leading to the upregulation of E2F target genes including cyclin E [15]. Then, cyclin E pairs up with its kinase partner, CDK2, to complete RB phosphorylation, which enables DNA replication and facilitates S-phase entry. Meanwhile, the CDK4/6–cyclin D1 complex also sequesters the endogenous CDK inhibitors such as p21 (also known as p21 WAF1/Cip1) and p27 (also known as p27 KIP1) to prevent the activation of the cyclin E–CDK2 complex, thereby initiating a positive feedback loop [13].

However, abnormal activation of CDKs could result in uncontrolled cell proliferation, leading to tumorigenesis [16]. Indeed, dysregulation of the CDK4/6–cyclin D1 complex is commonly found in many malignancies [17]. For example, CDK4 has been hyperactivated in up to 90% of melanoma cases and occurs through various mechanisms, including mutation of CDK4 (R24C) [18,19]. Additionally, Lazarov et al. revealed that the co-expression of CDK4 with Ras could result in human epidermal tumorigenesis [20]. CDK6 is frequently upregulated in hematopoietic tumors and plays a critical role in acute myeloid leukemia (AML) and acute lymphoblastic leukemia [21]. Scheicher et al. found that CDK6 binds to the promoter region of the FLT3 gene and PIM1 pro-oncogenic kinase, stimulating their expression in AML cells [22]. Similarly, the amplification of the CCND1 gene sequence or overexpression of cyclin D1 also induces cancer cell proliferation and tumorigenesis, which is frequently found in a higher proportion of human breast cancers and serves as a negative prognostic marker [23]. Thus, targeting the abnormally activated CDK4/6–cyclin D1 complex has been demonstrated to be a promising therapeutic approach against some cancers.

### 2.2. Therapeutic Strategies and Dilemma of CDK4/6 Inhibitors

**Figure 1:**
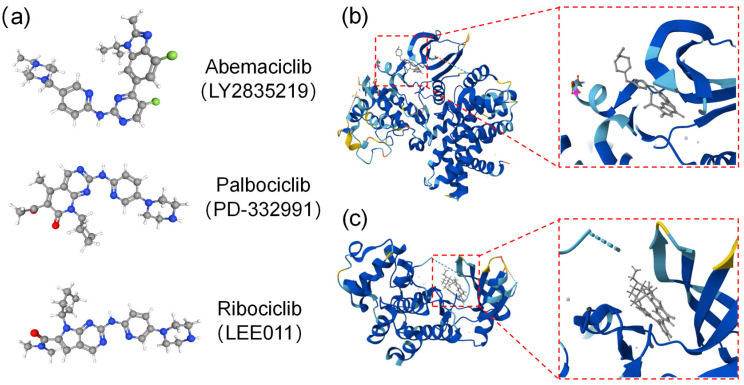
The structure of three CDK4/6 inhibitors and binding modes for abemaciclib with CDK4/6 kinases. (**a**) Chemical structures of CDK4/6 selective inhibitors (e.g., abemaciclib, palbociclib, and ribociclib). (**b**,**c**) Binding modes for abemaciclib: CDK4 (PDB code:7SJ3) (**b**) and CDK6 (PDB code:5L2S) (**c**) kinases’ co-crystal structures for abemaciclib, with the ATP-binding pocket located between the C- and N-terminal lobes.

Fortunately, numerous inhibitors targeting CDK4/6 kinases have exhibited great anticancer effects in several preclinical models and clinical trials over the past three decades. The initially designed pan-CDK inhibitors have shown a lack of specificity for CDKs and demonstrated significant toxicity during clinical trials in solid tumor therapy [24,25]. In light of the development of first- and second-generation CDK inhibitors, third-generation CDK4/6is (selectively targeting CDK4/6) like palbociclib, ribociclib, and abemaciclib have achieved great success in clinics [26]. These specific small-molecule inhibitors can specifically target ATP-binding pockets located between the C- and N-terminal lobes of CDK4 and CDK6 (Figure 1) [27]. Among them, abemaciclib is the only CDK inhibitor approved as monotherapy for HR^+^/HER2^−^ advanced or metastatic breast cancer, while two other CDK inhibitors (i.e., palbociclib and ribociclib) have been approved in combination with endocrine therapy [28].

Currently, a few preclinical studies have indicated that the combination of CDK4/6is with other signaling pathway-targeted agents has demonstrated excellent synergistic anticancer effects (Table 1). Briefly, CDK4/6is combined with an MAPK/EER inhibitor, ulixertinib (BVD-523), could synergistically suppress the growth of pancreatic ductal adenocarcinoma (PDAC) cells by blocking compensatory upregulation of ERK and PI3K [29]. In addition, the dual blockade of PI3Ka and CDK4/6 not only significantly enhanced the induction of apoptosis, cell-cycle arrest, and tumor immunogenicity but also induced immunogenic cell death in human triple-negative breast cancer (TNBC) cells [30]. Similarly, the combination of the CDK4/6i palbociclib paired with olaparib (a PARP inhibitor) has dramatically enhanced the inhibitory effect on the growth of BRCAmut/TNBCs by activating the Wnt signaling pathway [31].

On the other hand, it is well known that chemotherapy lacks specific drug targets and has significant side effects. Thus, combining chemotherapy with other agents is considered to be a cornerstone of cancer therapy [13]. Recently, it has been indicated that the sequential administration of CDK4/6is after administering chemotherapeutic agents can help to impede cellular proliferation through cell-cycle arrest and induction of DNA damage in PDAC cells (Table 1) [54]. In addition, CDK4/6is have also been used as an adjuvant therapy to radiation therapy for the treatment of glioblastoma, and they were observed to provide a survival advantage in mice models (Table 1) [55].

In addition, with the burgeoning of immunotherapy, an increasing number of studies have started to focus on the synergistic function between CDK4/6is and immunotherapy as well (Table 1) [10]. In patients with HR^+^/HER2^−^ metastatic breast cancer (MBC), the combination of CDK4/6is and immunotherapy was identified to enhance and prolong the duration of the antitumor response, with a complete response rate of 31% [35]. Another study demonstrated that CDK4/6is induced a pro-inflammatory immune response in the tumor microenvironment. Furthermore, the combination of CDK4/6is and anti-PD1/PD-L1 therapy achieved superior tumor control compared to monotherapy in cases of poorly immune-infiltrated ovarian cancer [56]. In certain patients with immunotherapy-tolerant advanced non-small-cell lung cancer (NSCLC), the combination of CDK4/6 inhibitors and anti-PD-1 agents has shown unexpected clinical benefits [36]. However, it is essential to remain vigilant regarding the potential drug toxicity associated with combination therapy, including adverse effects such as hepatotoxicity [51].

The more important concern is that although CDK4/6is have achieved tremendous success in recent years, drug resistance remains an inevitable dilemma. Briefly, resistance to CDK4/6is is mainly attributed to two aspects, namely, intrinsic and acquired resistance mechanisms [57]. Among them, the biggest challenge is acquired resistance, which is related to the activation of other compensatory mitogenic signaling pathways, tumor metabolism, alteration of the tumor environment, and so on [7]. In particular, acquired resistance to CDK4/6is is suggested to be closely associated with modulation of the immune responses caused by CDK4/6is. Therefore, it is critical to elucidate the relationship between the CDK4/6–cyclin D1 complex and the tumor microenvironment components after CDK4/6i treatment, namely, how these inhibitors induce favorable immunotherapeutic effects, or the reverse, in patients.

## 3. Immunomodulatory Effects of CDK4/6 Inhibitors

In recent years, leveraging the immune system to treat cancer by inhibiting immune checkpoints such as CTLA4, PD-1, and PD-L1 has resulted in a significant breakthrough in the field of cancer treatment [58,59,60]. Interestingly, several studies have demonstrated that CDK4/6–cyclin D1 also regulates the function and development of immune cells in the tumor microenvironment. They found that the inhibition of CDK4/6 kinases contributes to increasing the expression of major histocompatibility complex (MHC) class I in tumors, modulating T-cell activation, induction of memory CD8^+^ T cells, and so on [61,62]. Considering the impact of CDK4/6 kinases on the immune system, there are numerous studies shedding light on why inhibition of CDK4/6 kinases affects antitumor immune responses [63]. In light of the above findings, we summarize and discuss the latest studies that focused on the immunomodulatory effects of CDK4/6is.

### 3.1. Increase in Tumor Cell Immunogenicity

**Figure 2:**
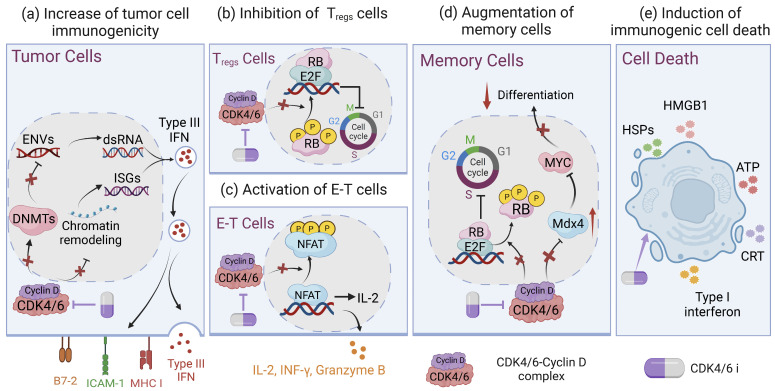
Immunomodulatory effects of CDK4/6 inhibitors. (**a**) CDK4/6is block the expression of DNMT1, increase levels of ENVs and dsRNA, induce the production of type III interferons, and upregulate the expression of MHC II, ICAM-1, and B7-2. Likewise, CDK4/6is also induce chromatin remodeling to regulate the ISG response and enhance antigen presentation. (**b**) In addition, regulatory T-cell (Treg) proliferation can be inhibited by CDK4/6is, (**c**) while effector T (E-T) cells can be activated by CDK4/6is by upregulating NFAT signaling. (**d**) CDK4/6is promote memory T-cell differentiation through inducing RB-mediated G1 arrest and increasing MXD4 gene expression, which mediated suppression of MYC, conferring the augmentation of memory potential in CD8^+^ T cells. (**e**) CDK4/6is suppress the phosphorylation of p73 and transcriptionally activate DR5, leading to immunogenic cell death of cancer cells. Created with Biorender.com.

Tumor immunogenicity, which refers to the ability of tumor cells to induce adaptive immune responses that can prevent tumor growth, is absolutely crucial for the effectiveness of immune response to therapies [64,65]. If the majority of solid tumors have low immunogenicity, this severely compromises the killing effect of immunotherapy, demonstrating that the low immunogenicity of tumor cells can make them prone to immune tolerance or immune escape at a later stage [66]. Therefore, multiple studies have attempted to enhance the immunogenicity of tumor cells through chemotherapy, radiotherapy, photoimmunotherapy, and other therapeutic approaches to improve the efficacy of immunotherapy [67,68,69]. Targeting CDK4/6 kinase therapy has several beneficial effects on enhancing tumor immunogenicity in various tumors, which include the induction of interferons (IFNs), increasing the expression of antigen-presenting molecules, and modulating the activation states of antigen-presenting cells (APCs), among other mechanisms (Figure 2a).

Chen and colleagues found that CDK4/6is potentiated the secretion of IFN-γ and the interferon-stimulated gene (ISG) response in ovarian cancer, which upregulated the expression of antigen-presenting molecules [70]. Furthermore, Goel et al. have elucidated how CDK4/6is enhance the immunogenicity of tumor cells in mouse models of breast cancer. One of the dominant mechanisms involves inhibiting the activity of DNA methyltransferases (DNMTs), which leads to increased expression of endogenous retroviral genes (ERVs) [63]. This regulation further controls the levels of double-stranded RNA, thereby stimulating the production of type III IFNs and upregulating MHC-I [71]. In addition, a recent study demonstrated that CDK4/6is induce chromatin remodeling characterized by the activation of multiple enhancers that directly overlap long terminal repeats (LTRs). This process facilitates the upregulation of ISGs and is predicted to regulate tumor immunogenicity [72]. Meanwhile, CDK4/6 kinase deficiency could trigger endogenous DNA damage, leading to the activation of cGAS-STING-dependent type I IFN production. This activation subsequently triggers IFNAR1 and induces ISG expression, enhancing tumor immunogenicity and antitumor immune responses [73].

David et al. also reported that abemaciclib could activate states of APCs such as dendritic cells (DCs) and macrophages, which enhance the multiple immune-related pathways including the Th1/2 pathway, indicative of an innate immune response and antigen presentation [74]. In certain tumors driven by Kaposi sarcoma-associated herpesvirus (KSHV) and Epstein–Barr virus (EBV), Wu and colleagues found that both viruses are able to evade detection by the human immune system through suppressing the expression of immune surface molecules such as major histocompatibility antigen class I (MHC-I), intracellular adhesion molecule 1 (ICAM-1), and B7-2 [75]. Conspicuously, CDK4/6is have a pronounced effect on enhancing the expression of these molecules and further inducing virus-specific immunity [75]. Taken together, the above observations indicate CDK4/6is are a promising strategy for enhancing tumor immunogenicity and strengthening the effectiveness of immunotherapy.

### 3.2. Increasing T-Cell Infiltration and Altering Myeloid Populations

T-cell activity is indispensable for the immune system’s functions and antitumor immunity [76]. Although CDK4/6is also have an antiproliferative effect on normal T cells by affecting cell cycle, short-term treatment with CDK4/6is could ultimately enhance antitumor immunity through increasing T-cell activity [61]. Recent studies have found that CDK4/6is have distinct effects on the immune system by increasing the infiltration of CD4^+^ and CD8^+^ T cells in various tumors, which is quite different from selectively blocking cell cycle [77,78]. Similarly, a study pointed out that inhibiting CDK4/6 kinases boosted the activity of the key transcription factor nuclear factor of activated T cells 1 (NFATC1; or NFAT), followed by the transcriptional regulation of T-cell mitogens (e.g., IL-2) and effector molecules (e.g., granzyme B), leading to the enhancement of effector T-cell functions in non-small-cell lung cancer xenograft mouse models (Figure 2c) [61,74]. In the murine ovarian cancer model, the CDK4/6i abemaciclib alone could enhance immune cell infiltration in tumors, particularly CD8^+^ T-cell and B-cell infiltration [56].

Moreover, Uzhachenko et al. found that the inhibition of CDK4/6 kinases leads to adoptive T-cell transfer or T-cell activation [78]. Mechanistically, CDK4/6is could recruit the activated CD8^+^ T cells through the induction of Th1 chemokines, including CCL5, CXCL9, and CXCL10 [78]. Likewise, Bai and colleagues reported that CDK4/6is upregulated intercellular adhesion molecule-1 (ICAM1) transcription by inhibiting the phosphorylation of RB protein, which enhances the homing and activation of adoptively transferred CD8^+^ T cells [79].

On the other hand, CDK4/6is are also equipped to downregulate several immunosuppressive T-cell populations (Figure 2b) [56,57]. Lai et al. elucidated that CDK4/6is exhibited a more potent proliferation inhibition effect on CD8^−^CD4^+^FOXP3^+^ regulatory T cells (Tregs) than effector T cells, resulting in an increased ratio of effector T cells to Tregs, which contributes to promoting effector T-cell function and triggering antitumor immunity [61,63]. In addition, CDK4/6is also reduced the presence of tumor-infiltrating immunosuppressive CD11c+ myeloid cells, which might be attributed to inhibiting the proliferation of bone marrow hematopoietic progenitors [61]. Collectively, CDK4/6 inhibition had a pronounced impact on enhancing the antitumor effect of immunotherapy drugs through increasing T-cell infiltration and altering myeloid populations. Nevertheless, it remains to be clarified whether such an immunostimulatory effect is caused by on-target effects on CDK4 and CDK6.

### 3.3. The Induction of Memory CD8^+^ T Cells

It is well known that immunologic memory is crucial for maintaining long-term immune surveillance after an infection has been eradicated. This memory enables the immune system to respond more rapidly and robustly to secondary infections [80,81]. The improvement of T-cell memory formation may present an appealing strategy to bolster cancer immunotherapy including CAR-T therapy [82,83,84].

Recent studies have highlighted the paramount role of CDK4/6is in skewing the CD8^+^ T cells towards memory cells through RB-dependent and RB-independent manners [80,81]. Firstly, it has been reported that CDK4/6is contribute to both the phenotypic and functional acquisition of memory CD8^+^ T cells by inducing RB-mediated G1 arrest and inhibiting CDK4/6 kinase signaling events [62]. This study also demonstrated that pretreatment of CAR-T cells with CDK4/6is significantly increases their T-cell persistence, long-term efficacy, and immunologic memory. Memory cell fate decisions are determined prior to the initial cell division, during which MYC is asymmetrically distributed between daughter cells. The daughter cell acquiring a higher concentration of MYC differentiates into an effector cell, whereas the daughter cell with a lower concentration of MYC is destined to become a memory cell [85]. Heckler et al. indicated that the inhibition of CDK4/6 upregulated a negative regulator of MYC, namely, Max dimerization 4 (MXD4), which mediated suppression of MYC, conferring the augmentation of memory potential in CD8^+^ T cells (Figure 2d) [86]. Although a limited number of studies investigated the effect of CDK4/6is on T-cell memory, it is undeniable that CDK4/6is influence T-cell memory fate commitment and these studies have shed new light on their clinical application, particularly in combination with immunotherapy.

### 3.4. The Induction of Immunogenic Cell Death

Immunogenic cell death (ICD) is a type of regulated cell death that can trigger an adaptive immune response in an immunocompetent milieu [87,88]. In the dying or stressed tumor cells, there are a series of molecules called damage-associated molecular patterns (DAMPs), which are recognized by innate pattern recognition receptors (PRRs) and can act as adjuvants or danger signals for the immune system [89]. Numerous studies have shown that chemotherapy, oncolytic viruses, radiotherapy, and photodynamic therapy are capable of inducing ICD by triggering various DAMPs, including the cell-surface translocation of calreticulin (CRT), high mobility group box 1 (HMGB1), heat-shock proteins (HSPs), extracellular release of ATP, and stimulation of type I interferon (IFN) responses. Eventually, these processes lead to the activation of tumor-specific immune responses [90,91].

Mounting evidence has shown that CDK4/6is can induce ICD to improve the anticancer efficacy of combination treatment (Figure 2e). In glioblastoma xenograft models, it has been demonstrated that a combination of CDK4/6is with oncolytic viruses promoted calreticulin exposure and extracellular ATP secretion, which indicated the enlargement of immunologic cell death in glioma cells [92]. Similarly, Teo et al. found that combining CDK4/6is with PI3Ka can increase the expression of tumor cell-surface calreticulin, which might further promote immunogenic cell death in TNBC [30]. Likewise, the combination of CDK4/6is with a HIF-2α inhibitor could promote ferroptosis, a type of immunogenic cell death associated with iron, by increasing ROS levels, suppressing SLC7A11 activity, and facilitating the release of HMGB1 and ATP in Merkel cell carcinoma [93]. Another report highlighted that CDK4/6is are capable of promoting ICD by transcriptionally activating death receptor 5 (DR5), a cytokine receptor and crucial component of the extrinsic apoptotic pathway, resulting in enhancing anticancer efficacy [94]. The above findings demonstrated that CDK4/6is might be able to improve the treatment efficacy of immunotherapy by inducing ICD in tumor cells.

## 4. Disorders of the Immune Environment in CDK4/6i-Resistant Cells

Indeed, the immunomodulatory effects of CDK4/6is are multifaceted and complex. Emerging studies suggest that tumor cells are capable of evading immune surveillance after being treated with CDK4/6is, potentially leading to drug resistance. Here, we discuss several paramount aspects regarding the resistance to CDK4/6is caused by immune dysregulation.

### 4.1. Aberrant Activation of the IFN Signaling Pathway

**Figure 3:**
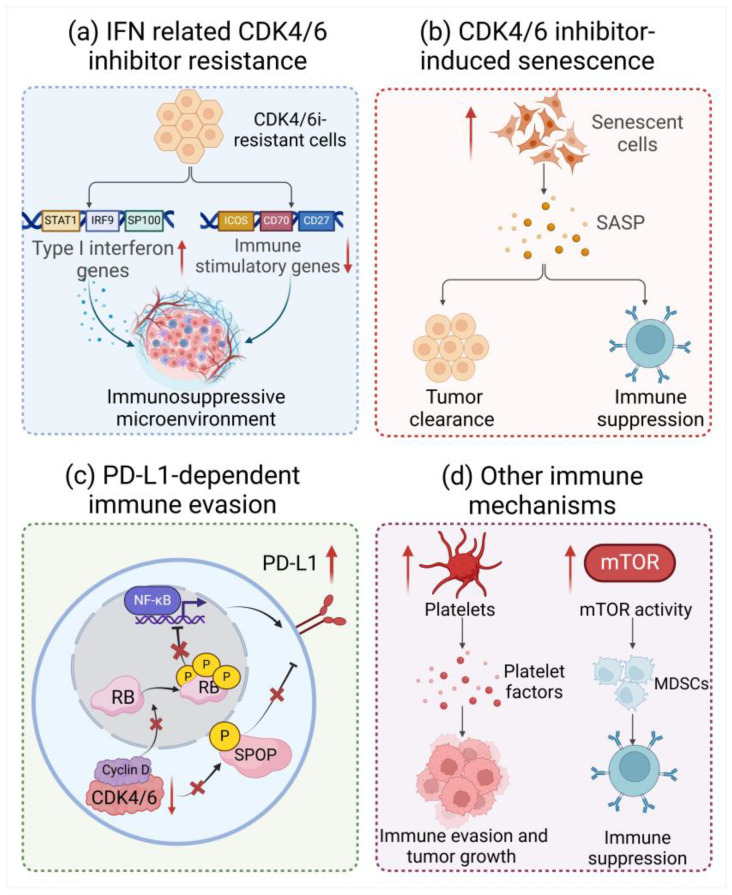
Disorders of immune environment in CDK4/6 inhibitor-resistant cells. (**a**) Type I IFN genes, including STAT1, IRF9, and SP100, were significantly upregulated, while immune stimulatory genes such as ICOS, CD70, and CD27 were suppressed in CDK4/6i-resistant cells. (**b**) CDK4/6is are capable of inducing cancer cell senescence and the secretion of SASP, which exerts paradoxical effects to facilitate tumor cell clearance or immune suppression. (**c**) CDK4/6is could enhance PD-L1 protein levels by reducing cullin3-SPOP ubiquitin ligase activity or suppressing hyperphosphorylation of RB. (**d**) CDK4/6is are found to increase platelet activity or mTOR activity, leading to immune evasion and tumor growth or an enhancement in the differentiation and recruitment of MDSCs. Created with Biorender.com.

IFNs (interferons) are a group of secreted proteins that activate the Janus kinase/signal transducer and the transcription (JAK/STAT) pathway, which leads to the expression of ISGs and further exhibits a significant role in host antiviral immunity [95,96]. Notably, IFNs have a double-faced effect that exerts either antitumor or protumor activity. For instance, IFNs are able to increase tumor cell immunogenicity (as described above), while the specific activation of IFNs can also transform allies into foes, conversely resulting in an immunosuppressive microenvironment. Currently, it has been indicated that aberrant activation of IFN signaling is associated with a reduction in cell sensitivity to CDK4/6is, leading to a poor prognosis in patients, which is referred to as the IFN-related palbociclib resistance signature (IRPS) [97]. IRPS is linked to an immunosuppressive tumor microenvironment characterized by activating forkhead box P3 (FOXP3^+^) CD4^+^ T lymphocytes, which further suppress T-cell functions and promote T-cell exhaustion [97,98]. Interestingly, Pandey et al. found that type I IFN genes, including STAT1, IRF9, and SP100, were significantly upregulated in MCF7 palbociclib-resistant cells, while immune stimulatory genes such as ICOS, CD70, and CD27 were suppressed in CDK4/6i-resistant cells [99]. Angelis et al. also found that “IFNα response” and “IFNγ response” were enriched gene signatures in intrinsically resistant breast cancer patients as well as cell lines. Moreover, IFN signaling is also significantly upregulated in breast cancer cells with acquired resistance to palbociclib [97]. In addition, Lypova et al. demonstrated that palbociclib-resistant breast cancer cells are probably associated with the change in type I IFN gene (i.e., IRF9) expression [100]. Thus, dysregulation of IFN signaling leads to resistance to CDK4/6is in breast cancer cell lines (Figure 3a). Additionally, blocking IFNγ may enhance CAR-T-cell function while reducing treatment-related toxicity in hematologic malignancies [101]. However, future studies are necessary to provide mechanistic insights into the relationship between IFN signaling and the response to CDK4/6is [102].

### 4.2. The Immunomodulation Effects of CDK4/6i-Induced Senescence

Cellular senescence is a state of stable cell-cycle arrest with complex biology and interplays with the immune system [103,104]. In multiple preclinical studies, CDK4/6is can induce the development of the senescence-like progress imposed by cell-cycle arrest in cancer cells [105,106,107]. Senescent cells primarily contribute to the immunostimulatory effects of CDK4/6is by releasing various soluble mediators, including cytokines, chemokines, growth factors, and proteases, which are known as the senescence-associated secretory phenotype (SASP) (Figure 3b) [108,109]. Strikingly, the immunomodulatory effects of the SASP vary considerably in different physiological milieus, which mainly depend on the molecular driver of senescence and the genetic characteristics of the tumor [110,111].

The cellular senescence induced by CDK4/6is exerts diverse effects on the tumor cells with various genetic configurations. For example, in non-malignant cells overexpressing p53, CDK4/6is exhibited effects by inducing p53-dependent senescence without NF-κB activity and NF-κB-driven SASP components. This type of senescence does not exhibit protumorigenic properties but retains the ability to promote paracrine senescence and facilitate cellular clearance [112]. Of note, although CDK4/6is have a beneficial effect on senescence in p53-proficient cells, prolonged treatment could also decrease p53 expression and prevent senescence. Guan et al. reported that prolonged treatment with CDK4/6is led to a decrease in Mdm2 and p53 protein levels in melanoma. This reduction suppressed the antitumor immune response and facilitated melanoma growth in immunocompetent cells [113]. Surprisingly, restoration or reactivation of p53 in cancer cells could accelerate senescence and tumor clearance, and increase therapeutic efficacy [113]. Similarly, inactivation or deficiency of p53 (i.e., mutant p53) in tumor cells could significantly affect the immunomodulatory effects of cellular senescence as well [114].

Another tumor suppressor protein, RB, also plays a significant role in maintaining the senescent state induced by CDK4/6is [115]. The integrity of the RB pathway is pivotal to the formation of senescence-associated heterochromatin foci (SAHF) which is a unique heterochromatic structure that accumulates in senescent human fibroblasts and stabilizes the state of senescence [116]. Wu et al. reported that interferon regulatory factor 3 (IRF3) can attenuate hyperphosphorylation of RB induced by CDKs and trigger cellular senescence by innate immune pathway cGAS-STING signaling [117]. Indeed, the immunomodulatory effects of cellular senescence induced by CDK4/6is are probably associated with the activity of tumor suppressor proteins p53 and RB [118].

### 4.3. PD-L1-Dependent Immune Evasion

The immune homeostasis and responses can be repressed by negative regulatory pathways, including programmed cell death protein 1 (PD-1) and its ligand PD-L1. PD-1 is widely expressed at the surface of immune cells such as activated T cells, and negatively regulates human immune response through binding with PD-L1, which is extensively expressed in antigen-presenting cells and tissues. The interaction of PD-1 with PD-L1 delivers inhibitory signals that are crucial for suppressing T-cell signaling, mediating tolerance mechanisms, and maintaining immune homeostasis. In addition, PD-L1 was found to be upregulated in cancer cells and could interact with PD-1 on tumor-infiltrating lymphocytes, which cause cancer immune escape. Mechanically, the binding of PD-L1 with PD-1 blocks the TCR signal cascade responses and its co-stimulatory signal transduction, inducing dysfunction as well as exhaustion of effector T cells. Moreover, it could also drive polarization of tumor-associated macrophages into the M2 type, promoting immunosuppression and cancer cell invasion by releasing cytokines [119,120,121,122]. A recent study identified that PD-L1 protein level can be repressed by cyclin D-CDK4 and E3 ligase cullin3-speckle-type POZ protein (SPOP) through proteasome-mediated degradation [123]. Therefore, inhibition of CDK4/6 kinases leads to an increase in PD-L1 protein levels by hindering cyclin D-CDK4-mediated SPOP, resulting in a decrease in the number of tumor-infiltrating lymphocytes (TILs) in tumors [123]. On the other hand, the knockdown of RB or treatment of CDK4/6is selectively upregulated a subset of NF-κB pathway genes including PD-L1. Zhi et al. also observed a significant increase in the expression of PD-1 TNBC after treatment with CDK4/6i. These results suggest that CDK4/6is may promote the stabilization of the PD-L1 protein, leading to tumor immune evasion and drug resistance (Figure 3c) [124].

Undeniably, targeting PD-1/PD-L1 immune checkpoints, known as immune checkpoint blockade (ICB) therapy, has been approved for treating human cancers with durable clinical benefits [125,126]. Currently, monoclonal antibodies that block PD-1/PD-L1 have been approved to treat various malignancies and have achieved immense success in clinical trials [127,128,129]. It is, however, insufficient to stimulate an effective antitumor immune response by blocking immune checkpoints in some patients [125]. One factor correlated with the response to these checkpoint agents is their expression levels in tumor cells [130]. It has been reported that innate resistance to anti-PD-1 immunotherapy is related to the genetic aberrations in the CDK4 pathway, including CDK4, CCND1 gain, and CDKN2A loss in patients with advanced melanoma [131]. In light of the role of CDK4/6is in increasing PD-L1 expression, there is potential for combining CDK4/6is with the PD-1/PD-L1 immune checkpoint blockade to improve therapeutic effectiveness in cancer treatment.

### 4.4. Other Immune Mechanisms of CDK4/6 Inhibitor Resistance

Currently, the role of platelets in promoting tumorigenesis is well recognized in the field of cancer biology. Platelets release factors that support tumor growth and also form heterotypic aggregates with tumor cells, which can provide an immune-evasive advantage [132]. Valenzuela et al. found that CDK4/6i-induced SASP could enhance the activity of platelets and promote platelet aggregation, which could accelerate the formation of an immunosuppressive microenvironment that promotes cancer progression [133]. Therefore, targeting platelet aggregation is probably a potential therapeutic strategy for reversing CDK4/6i resistance and restricting immune evasion (Figure 3d). In addition, mTOR signaling is also found to play a vital role in an immunosuppressive tumor environment [134]. Uzhachenko et al. reported that CDK4/6is are capable of promoting mTOR activity in mammary tumors; thus, inhibition of mTOR signaling might be a promising strategy in the context of CDK4/6i treatment (Figure 3d) [78]. In short, although it is now clear that cancer cells can evade immunosurveillance following CDK4/6i treatment through multiple mechanisms, more evidence is needed to demonstrate the universality of the above-mentioned phenomenon and the feasibility of treatment strategies.

## 5. Therapeutic Strategies and Risk Assessment for Using CDK4/6 Inhibitors in Clinics

Of note, clinical trials using a combination of CDK4/6is with immunotherapy have demonstrated promising potential for cancer patients. In patients with HR^+^/HER2^−^ MBC, Scirocchi et al. found that the decrease in regulatory T cells was associated with the clinical response to CDK4/6i treatment in vivo. Moreover, treatment with CDK4/6is also resulted in a reduction in the circulating myeloid-derived suppressor cell (MDSC) population (known as the queen bee of the tumor microenvironment) while preserving other T-cell subsets such as CD8^+^ and CD4^+^ T cells [135]. Likewise, a phase II trial in patients with metastatic triple-negative breast cancer confirmed that administration of CDK4/6is before chemotherapy could enhance T-cell activation and improve overall survival in patients [136]. Excitingly, there was a significant reversal in drug resistance after receiving intermittent treatment with CDK4/6is in a patient with advanced melanoma who exhibited resistance to immunotherapy [137].

On the other hand, it is worth noting that a series of clinical trials using CDK4/6is in combination with immune checkpoint inhibitors have demonstrated that there are challenges related to their adverse effects. For instance, in a phase 1b study of NSCLC, Pujol et al. warned that patients treated with a CDK4/6i (abemaciclib) and an immune checkpoint inhibitor (pembrolizumab) simultaneously tended to develop a higher rate of transaminase elevations and pneumonitis compared with previous reports [138]. Subsequently, Rugo et al. also pointed out that a higher rate of transaminase could be induced in patients with locally advanced or metastatic breast cancer by abemaciclib in combination with pembrolizumab [50]. These adverse effects are considered to be the result of an on-target effect by CDK4/6 inhibition [139]. Consequently, it is imperative and paramount for researchers to figure out the toxicity and efficacy issues of combining CDK4/6is with immunotherapy.

To this end, there is a growing body of research aimed at mitigating the risk of toxicity associated with the combination of CDK4/6is and immunotherapy. One feasible strategy is to reduce the concentration and duration of CDK4/6i. Excitingly, Charles et al. reported that low-dose CDK4/6is could induce the presentation of pathway-specific MHC ligands on breast cancer cell surfaces as potential targets for immunotherapy, suggesting that low-dose CDK4/6is can be an inducer for MHC ligand expression to improve the outcomes of patients receiving immunotherapy [140]. Lelliott et al. found that pretreatment of patients with CDK4/6is can enhance the persistence of functional memory CD8^+^ T cells and CAR-T cells [62]. Therefore, CDK4/6is could be used as a pretreatment drug instead of in combination with immunotherapy to reduce the risk of toxicity in clinics.

## 6. Conclusions and Future Perspectives

Over the past three decades, the emergence of CDK4/6is has been a significant milestone and achieved great success in the treatment of advanced breast cancer. However, drug resistance remains a major challenge in clinical settings. Until now, there have been no effective drugs available for CDK4/6i-resistant patients. Therefore, understanding the molecular mechanisms underlying CDK4/6i resistance is crucial for developing new therapeutic strategies. Accumulating evidence has pointed out that CDK4/6is not only regulate cell cycle but also exhibit an immune-priming effect on both tumor cells and immune cells. However, the immunological effects of CDK4/6is are contradictory. In malignant tumors characterized by low immunogenicity, CDK4/6is can enhance tumor cell immunogenicity by increasing the expression of antigen-presenting molecules and modulating antigen-presenting cell activation. Furthermore, CDK4/6is can induce immunogenic cell death in tumor cells through the activation of tumor-specific immune responses. By targeting the body’s immune system, CDK4/6is have been shown to bolster antitumor immunity by enhancing T-cell activity and promoting the activation of memory CD8^+^ T cells. Conversely, it may also lead to immune dysregulation that enables tumor cells to develop drug resistance. These mechanisms include abnormal activation of interferon signaling pathways, cellular senescence, and increased expression of PD-L1. Future studies are warranted to further explore the antitumor activity for combinations of immunotherapy with CDK4/6is in order to better elucidate how cancer cells evade immune surveillance following treatment with CDK4/6is and identify potential strategies for overcoming this resistance. These mechanistic investigations suggest that combining CDK4/6 inhibitors with immunotherapy holds significant promise for improving outcomes in cancer patients. This review delves into the immunomodulatory effect of CDK4/6is and the clinical challenges of combining CDK4/6is with immunotherapy, aiming to propose new therapeutic strategies to combat drug resistance. We have gathered compelling data to demonstrate that the use of CDK4/6is could alter the tumor immune microenvironment and further elucidate the association between CDK4/6i resistance and its regulatory effect of the immune environment. Importantly, it is necessary to explore the most suitable dosing and timing schedule for the combination of CDK4/6is and immunotherapy to maximize therapeutic efficacy and overcome drug resistance in patients.

## Figures and Tables

**Table 1:** Clinical studies of combination therapies containing CDK4/6 inhibitors.

CDK4/6 Inhibitor	Combinations	Treatments	Disease	Stage	Trial Identifier	References
Palbociclib	ERK MAPK inhibitor	Palbociclib and ulixertinib	KRASmutant pancreatic cancer	I	NCT03454035	[29]
	EGFR mAb plus radiotherapy	Palbociclib plus cetuximab and radiotherapy	Locally advanced head and neck squamous cell carcinoma	I	NCT03024489	[32]
	Chemotherapy	Palbociclib, and standard four-drug re-induction chemotherapy	Relapsed/refractory B- and T-cell lymphoblastic leukemia and lymphoma	I	NCT02255461 NCT03792256	[33]
	IGF-1R monoclonal antibody	Palbociclib and ganitumab	Ewing sarcoma	II	NCT04129151	[34]
	Immune checkpoint inhibitor and aromatase inhibitor	Palbociclib, pembrolizumab, and letrozole	Metastatic breast cancer	I/II	NCT02778685	[35]
	Anti-PD-L1 immunotherapy and anti-VEGFR	Avelumab, palbociclib, and axitinib	Advanced non-small-cell lung cancer	I	NCT03386929	[36]
	EGFR inhibitor and PD-L1 inhibitor	Cetuximab and avelumab	Head and neck squamous cell carcinoma	I	NCT03498378	[37]
	Immune checkpoint inhibitor	Palbociclib and PD-1 inhibitor	Metastatic rare solid tumors	II	NCT04423185	[38]
Ribociclib	Aromatase inhibitor	Letrozole	Advanced or metastatic breast cancer	IIIb	NCT03096847	[39]
	Aromatase inhibitor and tyrosine kinase inhibitor	Exemestane, ribociclib, and everolimus	Advanced breast cancer	I/II	NCT02732119	[40]
	Chemotherapy	Doxorubicin, cyclophosphamide, and paclitaxel plus ribociclib	Luminal B breast cancer	II	NCT03248427	[41]
	Chemotherapy	Docetaxel plus ribociclib	Metastatic castration-resistant prostate cancer	Ib/II	NCT02494921	[42]
	Endocrine therapy	Letrozole or anastrozole	Breast cancer	III	NCT03701334	[43]
	Immunotherapy	Everolimus	Intrinsic pontine glioma (DIPG) and high-grade glioma (HGG)	I	NCT02607124”	[44]
	Immunotherapy	Everolimus	Foregut neuroendocrine tumors	II	NCT03070301	[45]
	Tyrosine kinase inhibitor	Ribociclib and everolimus	Liposarcoma and leiomyosarcoma	II	NCIP30CA00692	[46]
Abemaciclib	Aromatase inhibitor	Letrozole	Ovarian cancer	II	NCT05872204	[47]
	PI3K/mTOR inhibitor or TGF-βRI inhibitor	LY3023414 or galunisertib	Metastatic pancreatic adenocarcinoma	II	NCT02981342	[48]
	Endocrine therapy	Lasofoxifene plus abemaciclib	Metastatic breast cancer	II	NCT04432454	[49]
	Immune checkpoint inhibitor	Pembrolizumab	Metastatic breast cancer	Ib	NCT02779751	[50]
	PD-L1 inhibitor and type II MET kinase inhibitor	LY3300054, ramucirumab, abemaciclib, or merestinib	Advanced, refractory solid tumors	Ia/Ib	NCT02791334	[51]
	Immune checkpoint inhibitor and endocrine therapy	Nivolumab and abemaciclib plus endocrine therapy	Metastatic breast cancer	II	WJOG11418B	[52]
	Tyrosine kinase inhibitor	Lenvatinib	Hepatocarcinoma	II	NCT03781960	[53]
